# Knowledge, attitudes, behavior, and self-efficacy related to evidence-based practice among healthcare professionals working in the municipal healthcare service in Norway: a cross-sectional survey

**DOI:** 10.1186/s12913-024-11723-4

**Published:** 2024-10-15

**Authors:** Nils Gunnar Landsverk, Nina Rydland Olsen, Therese Brovold

**Affiliations:** 1 Department of Rehabilitation Science and Health Technology, Faculty of Health Science, Oslo Metropolitan University, Oslo, Norway; 2 Faculty of Health and Social Sciences, Department of Health and Functioning, Western Norway University of Applied Sciences, Bergen, Norway

**Keywords:** Evidence-based practice, Healthcare professional, Knowledge, Attitudes, Self-efficacy, Behavior

## Abstract

**Background:**

Practicing the process of evidence-based practice (EBP) may be challenging for healthcare professionals and may be affected by their EBP knowledge, attitudes, self-efficacy, and behavior. We have some insight into how Norwegian healthcare professionals and students perceive EBP. However, research on the perception of EBP among primary healthcare professionals working in the Norwegian municipal health service is lacking. This study aimed to map EBP knowledge, attitudes, behavior, and self-efficacy among healthcare professionals working with older people in the municipal health service in Norway and to examine associations between how they score and their background characteristics.

**Methods:**

A cross-sectional web-based survey was conducted among healthcare professionals in the Norwegian municipal healthcare service. We used the revised Norwegian version of the Evidence-based practice profile questionnaire (EBP^2^-N) to measure the healthcare professionals’ EBP knowledge, attitudes, behavior, and self-efficacy, operationalized through the five domains of the EBP^2^-N. We calculated the mean scores for each EBP domain across the total sample and for each subgroup of healthcare professionals. We used a one-way between-groups analysis of variance (ANOVA) to analyze the differences in mean scores between the professions. We also calculated eta-squared values to determine effect size. We used linear regression analyses to examine associations with background variables.

**Results:**

A total of 313 healthcare professionals, including nurses, assistant nurses, physical therapists, occupational therapists, and medical doctors, responded to the survey. The total sample scored the highest on the *relevance* domain, with a mean domain score of 58.9 (95% CI = 58.1–59.7) on a scale ranging from 14 to 70. The *practice* domain had the lowest score, with a mean domain score of 22.2 (95% CI = 20.8–21.6) on a scale ranging from 9 to 45. Statistically significant differences in mean scores were found between professions in all domains except the *confidence* domain. The most considerable differences between professions’ mean scores were found for the *relevance* and *terminology* domains, with eta-squared values of 0.13 and 0.19, respectively. The multivariate regression results showed that EBP training was significantly associated with the sum score of the *relevance*, *terminology*,* and confidence* domain. However, EBP training was not associated with the sum score of the *practice* and *sympathy* domains.

**Conclusions:**

Primary healthcare professionals in the Norwegian municipal healthcare service hold positive attitudes toward EBP. However, they report a low understanding of research terms, low self-efficacy in performing EBP activities, a lack of perceived compatibility of EBP with professional work, and a low frequency of EBP behavior. Additionally, we observed differences among the included professions in four out of five domains, suggesting that various professions may be unequally prepared for EBP. Finally, our results indicate potential positive outcomes of EBP training. Those who received EBP training exhibited more positive attitudes, a better understanding of common research terms, and higher self-efficacy in performing EBP activities. However, EBP training was not associated with their self-reported EBP behavior.

**Trial registration:**

Retrospectively registered (prior to data analysis) in OSF Preregistration. Registration DOI: 10.17605/OSF.IO/428RP.

**Supplementary Information:**

The online version contains supplementary material available at 10.1186/s12913-024-11723-4.

## Background

Evidence-based practice (EBP) requires clinicians to use their clinical expertise to integrate information from research evidence and patient perspectives to assist patients in making the most optimal decision within their specific context [1, p. 16–18, 2, 3]. The EBP process comprises five steps: ask, search, appraise, integrate, and evaluate [2, 3, p. 4–5]. Applying EBP is a challenging process prone to individual, organizational, social, and economic barriers [4–6]. At the individual level, factors such as EBP knowledge and skills, attitudes toward EBP, and self-efficacy may influence EBP behavior [5–9]. Practicing the different EBP steps requires that the clinician hold a set of EBP core competencies [10]. Clinicians need to understand the concepts of EBP, including the EBP process, basic principles, relevant terms, and levels of evidence (EBP knowledge) [2, 11]. Additionally, healthcare professionals’ confidence in their ability to perform EBP activities (self-efficacy) and their beliefs in the importance and usefulness of EBP (attitudes) are known to be associated with successful implementation of EBP in clinical practice (behavior) [8, 9, 11].

Measuring factors such as EBP knowledge, attitudes, self-efficacy, and behavior may help healthcare organizations clarify performance expectations, which in turn may be used to guide professional practice toward evidence-based clinical decision-making [12]. Strategies to change the clinical behavior of healthcare professionals towards EBP may be more likely to succeed if they are based on the factors known to affect the target behavior [13]. Additionally, we know that EBP training, education level, work experience, and age may be associated with healthcare professionals’ self-reported EBP knowledge, attitudes, self-efficacy, and behavior [9, 14–17]. Therefore, it could also be helpful to collect information on the healthcare worker’s background characteristics when developing EBP implementation strategies.

Current international research literature on healthcare professionals’ EBP competencies, such as knowledge, skills, attitudes, beliefs, and implementation, was summarized and synthesized in an overview of systematic reviews published in 2019 [8]. This umbrella review, which included 11 systematic reviews published between January 2012 and July 2017, included 59,321 healthcare professionals from Europe, Australia, Asia, South America, and North America [8]. The authors found that most healthcare professionals were familiar with the term EBP. However, many were confused and lacked knowledge about basic definitions and concepts related to EBP. Further, they found that although most healthcare professionals held positive attitudes and beliefs related to the importance of EBP, many reported a lower level of perceived EBP knowledge and skills and even lower levels of EBP behavior [8]. The authors of the umbrella review concluded: “Large proportions of practicing healthcare professionals perceive their EBP competencies to be insufficient for employing EBP in daily care delivery” [8].

In Norway, the health authorities have focused on developing EBP competence and ensuring evidence-based services in municipal healthcare [18–21]. Several studies have investigated the status of Norwegian healthcare professionals’ EBP knowledge, attitudes, self-efficacy, behavior, and other factors known to affect EBP implementation [22–25]. Stokke et al. (2014) found that nurses in specialized healthcare held positive attitudes toward EBP but only practiced evidence-based to a small extent [24]. Moore et al. (2018) reported that healthcare professionals in public hospitals and private rehabilitation centers regularly did literature searches and critical appraisals [23]. However, the same healthcare professionals reported a lower degree of integration of research evidence into clinical practice compared to search and appraisal [23]. Egeland et al. (2016) reported positive attitudes toward EBP in mental healthcare professionals in both primary and specialized healthcare, although primary healthcare professionals scored significantly higher [25]. Lastly, Snibsøer et al. (2018) found that bachelor students in different professions held positive attitudes toward EBP. However, the students reported low EBP knowledge, self-efficacy, and use of EBP in clinical situations [22]. Regarding associations with background characteristics, Stokke et al. (2014) found that healthcare professionals who had received EBP training held more positive beliefs about EBP [24]. Olsen et al. (2014) found that a higher level of EBP exposure in third-year physical therapist students was associated with a better perceived ability to practice evidence-based during clinical placements, such as critically appraising research evidence and searching for research in databases [26]. Moore et al. (2018) found a small positive association between academic degree and EBP implementation [23], while Egeland et al. (2016) found that more work experience and older age were associated with more negative attitudes towards EBP [25].

The studies above offer insight into how Norwegian healthcare professionals and students perceive EBP and the potential associations with background characteristics and EBP factors such as knowledge, attitudes, self-efficacy, and behavior. However, we still need to gain knowledge of these factors among primary healthcare professionals working in the Norwegian municipal health service. At the same time, the Norwegian government’s white paper on the National Health and Collaboration Plan (Meld. St. 9 (2023–2024)) emphasizes that services must be evidence-based to provide patients and users with the best possible care [19]. To meet these demands for evidence-based primary healthcare, we conducted a study to comprehensibly map the status of self-reported levels of EBP knowledge, attitudes, self-efficacy, and behaviors among primary healthcare professionals in the Norwegian municipal healthcare service. Specifically, our objectives were to: (1) map EBP knowledge, attitudes, behavior, and self-efficacy among primary healthcare professionals working with older people in the municipal health service in Norway, (2) examine the associations between the healthcare professionals’ background characteristics and their self-reported EBP knowledge, attitudes, self-efficacy, and behavior.

## Methods

### Study design

A cross-sectional web-based survey study was conducted among primary healthcare professionals working in the Norwegian municipal healthcare service. The study was reported following the Strobe checklist [27], and was pre-registered in the OSF registries prior to data analysis [28].

### Recruitment and participants

The study sample was recruited from Norwegian primary healthcare professionals working with older people, including physical therapists, occupational therapists, nurses, assistant nurses, and medical doctors. Proficiency in reading and understanding Norwegian was a prerequisite for inclusion. A snowball sampling approach was employed to recruit participants. An invitation letter, with details of the study and a consent form, was sent via e-mail to healthcare managers across more than 37 cities and municipalities spanning the eastern, western, central, and northern parts of Norway. The managers then passed on the invitation to eligible healthcare professionals and encouraged them to complete the questionnaire. Those who consented to participate were automatically provided a link to the online survey.

### Data collection procedures

We conducted a web-based cross-sectional survey using the survey solution nettskjema.no, developed and hosted by the University of Oslo [29]. Data collection occurred from October 2022 to June 2023.

### Measurement

We used a revised Norwegian version of the Evidence-based practice profile questionnaire (EBP^2^-N) [30, 31] to collect data in this study. The EBP^2^ was initially developed by McEvoy et al. in 2010 for healthcare professionals and students in Australia [31] and translated into Norwegian by Titlestad et al. in 2017 [30]. The EBP^2^-N is a self-reported questionnaire measuring healthcare professionals’ EBP knowledge, attitudes, behavior, and self-efficacy. The questionnaire is divided into five domains: r*elevance*,* sympathy*,* terminology*,* practice* and *confidence*. It consists of 58 items, which are rated on a 5-point scale. The questionnaire is multifactorial, and each domain is treated as a separate subscale and summarized, with higher scores indicating a higher degree of the construct measured in the domain in question. The full version of the original EBP^2^ has previously been published [32].

Below is a description of the five domains of the EBP^2^ [31] and their relation to the constructs of interest in this study: EBP knowledge, attitudes, behavior, and self-efficacy. The *relevance* domain (14 items) measures the value, emphasis, and importance individuals’ place on EBP. This domain includes items such as “EBP improves the quality of my work” and “The use of EBP is necessary in my work” [31]. This domain is related to the construct of EBP attitudes. The *sympathy* domain (7 items) measures the perceived compatibility of EBP with professional work [31]. It includes items such as “EBP does not take into account my clients’ preferences”, suggesting that the *sympathy* domain is also related to EBP attitudes. However, because it is more specifically connected to the practical fit of EBP in the work environment, the *sympathy* domain measures slightly different components of EBP attitudes compared to the *relevance* domain. The *terminology* domain (17 items) measures the perceived understanding of common research terms. It includes items asking clinicians to rate their understanding of terms, such as “systematic review”, “intention to treat”, and “treatment effect size” [31]. This domain relates to the part of the EBP knowledge definition described in the background, which involves understanding relevant terms. The *practice* (9 items) domain measures the use of EBP in clinical practice. It includes items asking how often clinicians practice EBP behaviors, such as searching for, reading, and appraising research evidence, as well as considering patient preferences [31]. This domain is related to the EBP behavior construct. The *confidence* domain (11 items) measures an individual’s perception of their EBP skills. It includes items on the clinician’s confidence in performing various EBP activities [31]. This domain is related to the self-efficacy construct.

According to results from a recent systematic review [33], the EBP^2^ questionnaire had overall sufficient content validity and internal consistency and reliability were sufficient in all domains except for the *sympathy* domain. These results were based on five different studies included in the review [30, 31, 34–36]. Regarding structural validity, the results of the review were inconsistent, and the five-factor model could not be confirmed [33]. The Norwegian version of the EBP^2^ (EBP^2^-N) was cross-culturally adapted and psychometrically tested in a sample of healthcare bachelor students in the study by Titlestad et al. (2017). Titlestad et al. found the questionnaire valid and reliable for the *relevance*, *terminology*, and *confidence* domains. However, they could not confirm the original five-factor model using a confirmatory factor analysis, and they also recommended further linguistic improvements to the Norwegian version [30]. Based on recommendations to further study the psychometric properties of the EBP^2^-N [30, 33], we comprehensibly assessed the content validity, structural validity, and internal consistency of the EBP^2^-N in a sample of Norwegian primary healthcare professionals [37]. The qualitative content validity interviews in this study revealed positive perceptions of the content validity of the EBP^2^-N, though with nuanced concerns about the relevance and comprehensibility of certain items. Additionally, a confirmatory factor analysis (CFA) showed uncertainty regarding the five-factor structure of the EBP^2^-N [37]. Minor linguistic revisions to some items made the questionnaire more understandable. We concluded that the EBP^2^-N was suitable for measuring Norwegian primary healthcare professionals’ EBP knowledge, attitudes, self-efficacy, and behavior. However, the results of the terminology domain should be interpreted with caution [37].

### Sociodemographic characteristics

In addition to the fifty-eight domain items, the questionnaire included demographic characteristics of the participants, such as age, profession, level of education, years since education, clinical work experience, and EBP training.

### Statistical methods

Statistical analyses were performed using IBM SPSS Statistics version 28 [38]. Descriptive statistics were applied to identify the background characteristics of the total sample. Continuous data were presented as mean and standard deviation (SD) if normally distributed. Categorical data were presented as frequencies (n) and percentages (%). The domain scores were calculated for each domain for the total sample and each subgroup of healthcare professionals. Due to the varying numbers of items per domain, the maximum domain scores varied (*relevance* 70, *terminology* 85, *confidence* 55, *practice* 45, and *sympathy* 35). The scores of the *Sympathy* domain were reversed before analysis due to negatively phrased items.

We analyzed the differences in mean domain scores between the professions, using a one-way between-groups analysis of variance (ANOVA). We calculated eta squared values to determine effect size, using the following threshold values: small (0.01), medium (0.06), and large (0.14)^39^, p. 260]. Post hoc comparisons were conducted to explain differences in the domains in cases where the ANOVA analysis showed statistically significant differences in mean scores between professions [39, p. 255–261]. Univariate linear regression was used to test whether EBP training, education level, number of years since finished education and professional training were associated with the domain scores. Multivariate linear regressions were conducted to evaluate the relative contribution of each background variable’s associations with the domain scores, adjusted for age. Preliminary analyses were conducted to check whether linear regression assumptions were met.

### Sample size calculation and handling of missing data

Regarding sample size, the power analysis showed that to detect a standardized mean difference (Delta 0.3–0.5) between subgroups of healthcare professionals, 64–173 participants were required for each group (sig 5%, power 80%). For multiple regression, > ten participants per variable were required in the analysis [39, p. 151, 40]. Participants with more than 25% missing data in the domain items were excluded from subsequent analyses. Individuals with over 20% missing data in a specific domain were omitted from the analysis of that specific domain only. Finally, for participants with 20% or less missing data on a particular domain, the missing values were replaced with the mean of the participant’s responses to other items within the same domain, given that data was assumed to be missing completely at random [41]. Little’s MCAR test was conducted to determine whether data were randomly missing [42].

### Ethical approval and consent to participate

We obtained written informed consent from the cross-sectional survey participants. The Norwegian Agency for Shared Services in Education and Research (SIKT) approved the study in March 2022 (ref: 747319).

## Results

### Participants

Table 1 outlines the characteristics of the 313 study participants included in the study. The participant’s mean age (SD) was 42.7 years (11.4). The sample comprised 119 nurses, 74 assistant nurses, 64 physical therapists, 38 occupational therapists, three medical doctors, and 15 other professionals, predominantly social educators (n = 8). Over half the participants (63.9 %) had bachelor’s degrees, 11.8 % had master’s degrees, 0.3 % had a Ph.D., 10.5 % had completed upper secondary education, and 13.1 % had attained tertiary vocational education. Notably, 38.8 % had completed their most recent education within the last five years, and 55 % had over ten years of clinical work experience. More than half of the participants (59.1%) had not received formal EBP training. Among the 128 participants who had undergone formal EBP training, 31.5 % had completed more than 20 hours of EBP training. Characteristics of study participants per healthcare profession are presented in Additional file 2. 

**Table 1:** Characteristics of study participants (*n*= 313)

	**n (%)**	**Mean (SD)**
**Age (years) (*n*** **= 291)**		42.7 (11.4)
**Profession (*n*** **= 313)**		
Occupational therapist	38 (12.1)	
Physical therapist	64 (20.4)	
Nurse	119 (38.0)	
Assistant nurse	74 (23.6)	
Medical doctor	3 (1.0)	
Other	15 (4.8)	
**Level of education (*n*** **= 313)**		
Below upper secondary education	1 (0.3)	
Upper secondary education	33 (10.5)	
Tertiary vocational education	41 (13.1)	
Bachelor’s Degree	200 (63.9)	
Master’s Degree	37 (11.8)	
PhD	1 (0.3)	
**Years since education (*n*** **= 313)**		
0–5 years	121 (38.7)	
6–10 years	57 (18.2)	
11–15 years	50 (16.0)	
16–20 years	24 (7.7)	
21–25 years	30 (9.6)	
26–30 years	17 (5.4)	
Over 30 years	14 (4.5)	
**Clinical work experience (*n*** **= 313)**		
0–5 years	68 (21.7)	
6–10 years	73 (23.3)	
11–15 years	46 (14.7)	
16–20 years	38 (12.1)	
21–25 years	34 (10.9)	
26–30 years	19 (6.1)	
Over 30 years	35 (11.2)	
**EBP experience/ training (No)**	185 (59.1)	
**Yes**	128 (40.7)	
1–3 h	14 (11.3)	
3–10 h	45 (36.3)	
10–20 h	26 (21.9)	
Over 20 h	39 (31.5)	

Other = social educators, assistants, leaders

### Missing data

A total of 314 participants responded to the questionnaire. The Little’s MCAR test showed p-values higher than 0.05 for all domains, indicating that data was missing completely at random. For the 26 participants with less than 20% missing data in a specific domain, the missing values were substituted with the mean of the same domain. One participant was excluded from further analyses due to having more than 25% missing data in the domain items, leaving a total sample of 313. Additionally, two participants had over 20% missing data on the domain items, both had missing data in the *confidence* domain and one in the *practice* domain. Data from these two participants were omitted from the analyses of the *confidence* and practice domains only. The maximum percentage of missing data on one domain item was 1.3%. The percentage of missing data per domain was low (*relevance* = 0.05%, *sympathy* = 0.2%, *terminology* = 0.4%, *practice* = 0.6%, *confidence* = 0.6%). The only background variable with missing data was “age,” with 7% missing data. When visually inspecting the age variable, we found no missing data pattern, and the missing data seemed to spread evenly across the professions and education levels. Missing data on age were not substituted with new values, which explains the lower number of participants (*n* = 291) in the multiple regression analyses.

### Scores on EBP2-N

The mean domain scores for the total sample on the EBP^2^-N are presented in Table 2. The total sample (*n* = 313) scored the highest on the *relevance* domain, with a mean domain score of 58.9 (95% CI = 58.1–59.7) on a scale ranging from 14 to 70. The *practice* domain had the lowest score, with a mean domain score of 22.2 (95% CI = 20.8–21.6) on a scale ranging from 9 to 45. Mean domain scores per healthcare profession are presented in Table 3.

**Table 2:** EBP2-N domain scores for the total sample (*n* = 313). Differences between the profession’s mean scores were tested using one-way ANOVA (*n* = 295)

EBP^2^-*N* subscales (min-max values)	Domain score Mean (95% CI)	One-way ANOVA ^1^ F, *p*-value, eta squared (η2)
Relevance (14–70)	58.9 (58.1–59.7)	F(3, 291) = 14.3, *p* < 0.001, η2 = 0.13
Terminology (17–85)	44.5 (42.8–46.2)	F(3, 291) = 22.8, *p* < 0.001, η2 = 0.19
Confidence (11–55)	31.2 (30.1–32.2)	F(3, 289) = 2.6, *p* = 0.055, η2 = 0.03
Practice (9–45)	22.2 (21.5–22.8)	F(3, 290) = 5.1, *p* = 0.002), η2 = 0.05
Sympathy (7–35)	21.2 (20.8–21.6)	F(3, 291) = 7.8, *p* < 0.001, η2 = 0.07

^1^One-way ANOVA was tested on physical therapists (*n* = 64), occupational therapists (*n* = 38), nurses (*n* = 119), and assistant nurses (*n* = 74), in total *n* = 295. Data was missing in two domains: *Practice n* = 1, *Confidence n* = 2

**Table 3:** EBP2-N domain scores per healthcare profession

	EBP^2^-*N* domain scores				
**Healthcare profession**	**Relevance** **(14–70)** Mean (SD)	**Terminology** **(17–85)** Mean (SD)	**Confidence** **(11–55)** Mean (SD)	**Practice** **(9–45)** Mean (SD)	**Sympathy** **(7–35)** Mean (SD)
Physical therapists (*n* = 64)	62.7 (5.4)	55.0 (13.1)	33.1 (9.5)	24.1 (4.5)	22.8 (3.5)
Nurses (*n* = 119)	59.6 (6.8)	44.1 (14.5)	31.9 (9.6)	22.6 (5.9)	20.8 (3.9)
Assistant nurses (*n* = 74)	55.3 (7.9)	35.5 (13.4)	29.3 (9.0)	20.5 (6.6)	20.0 (3.0)
Occupational therapists (*n* = 38)	58.5 (5.9)	44.0 (13.4)	29.7 (7.8)	21.2 (4.7)	21.2 (3.4)
Medical doctors (*n* = 3)	57.7 (6.7)	56.7 (16.7)	26.7 (11.0)	23.0 (6.0)	22.0 (4.0)
Other (*n* = 15)	56.3 (9.4)	46.8 (14.6)	30.2 (8.5)	20.1 (4.9)	23.1 (3.2)

“Other” = Social educators, assistants, leaders

### Differences between professions

The one-way ANOVA included physical therapists, occupational therapists, nurses, and assistant nurses, in total 295 participants. The analysis revealed at least one statistically significant difference in mean scores between physical therapists, occupational therapists, nurses, and assistant nurses in all domains except for *confidence* (Table 2). The most considerable differences between professions’ mean scores were found in the *relevance* and *terminology* domains, with eta-squared values of 0.13 and 0.19, respectively.

Post hoc analysis using the Tukey HSD test (Table 4) revealed that physical therapists scored statistically significantly higher than all the other professions on the *relevance* and *terminology* domain. The most considerable difference was found in the *terminology* domain, with physical therapists scoring 19.50 (95% CI 13.4–25.6) higher than assistant nurses. Assistant nurses also scored statistically significantly lower than the other professions in the *terminology* domain. In the *practice* domain, the only statistically significant difference in mean score was between physical therapists who scored higher than assistant nurses. In the *sympathy* domain, there were statistically significant differences in mean scores, with physical therapists scoring higher than nurses and assistant nurses. No statistically significant differences in mean scores were found between occupational therapists and nurses in any domain.

**Table 4:** Post hoc analysis of differences in mean scores between healthcare professions on the relevance, terminology, practice, and sympathy domains (*n* = 295)

*EBP* ^*2*^ *-N subscales (Min - max values)*				
**Relevance (14 - 70) (** ***n*** ** = 295)**				
**Healthcare professions**	**Mean difference**	***P*** **-value**		
			**Lower**	**Upper**
Physical therapists - Occupational therapists *	4.18	0.014	0.62	7.74
Physical therapists – Nurses *	3.07	0.018	0.38	5.77
Physical therapists - Assistant nurses *	7.39	< 0.001	4.43	10.36
Assistant nurses - Occupational therapists	-3.22	0.80	-6.68	0.25
Assistant nurses – Nurses *	-4.32	< 0.001	-6.89	-1.75
Nurses - Occupational therapists	1.10	0.82	-2.13	6.89
**Terminology (17–85) (*n*** **= 295)**				
**Healthcare professions**	**Mean difference**	***P*** **-value**	**95% CI**	
			**Lower**	**Upper**
Physical therapists - Occupational therapists *	11.04	< 0.001	3.73	18.36
Physical therapists – Nurses *	10.95	< 0.001	5.41	16.49
Physical therapists - Assistant nurses *	19.50	< 0.001	13.40	25.60
Assistant nurses - Occupational therapists *	-8.46	0.013	-15.59	-1.33
Assistant nurses – Nurses *	-8.55	< 0.001	-13.84	-3.27
Nurses - Occupational therapists	0.09	1.00	-6.56	6.75
**Practice (9–45) (*n*** **= 294)**				
**Healthcare professions**	**Mean difference**	***P*** **-value**	**95% CI**	
			**Lower**	**Upper**
Physical therapists - Occupational therapists	2.96	0.057	-0.06	5.97
Physical therapists – Nurses	1.52	0.31	-0.76	3.80
Physical therapists - Assistant nurses *	3.59	0.002	1.07	6.11
Assistant nurses - Occupational therapists	-0.64	0.94	-3.58	2.31
Assistant nurses – Nurses	-2.07	0.070	-4.26	0.11
Nurses - Occupational therapists	1.44	0.528	-1.30	4.18
**Sympathy (7–35) (*n*** **= 295)**				
**Healthcare professions**	**Mean difference**	***P*** **-value**	**95% CI**	
			**Lower**	**Upper**
Physical therapists - Occupational therapists	1.59	0.12	-0.27	3.46
Physical therapists – Nurses *	2.06	0.001	0.64	3.47
Physical therapists - Assistant nurses *	2.8	< 0.001	1.25	4.36
Assistant nurses - Occupational therapists	-1.21	0.32	-3.03	0.61
Assistant nurses – Nurses	-0.75	0.48	-2.09	0.60
Nurses - Occupational therapists	-0.46	0.90	-2.16	1.23

Post hoc analyses were conducted using the Tukey HSD test

*- The differences in means was significant at the 0.05 level

### Associations between background variables and scores on the different domains

Five multivariate regression models were calculated to evaluate the associations between background variables and the mean domain scores on *the relevance*,* terminology*,* confidence*,* practice*, and *sympathy* domains. In each of the five models, the following independent variables were included: level of education (coded: 1 = < bachelor’s degree, 2 = bachelor’s degree or higher), EBP training (coded: 1 = No, 2 = Yes), years since education (coded: 1 = 0–5 years, 2 = 6 years or more), professional training (dummy variables with assistant nurse as reference), and age (number of years). The unadjusted and adjusted estimates of the five models are presented in Tables 5, 6, 7, 8 and 9. Preliminary analyses were conducted to check whether any assumptions had been violated. The variable “Level of education (< bachelor’s degree / > bachelor’s degree)” was removed from all five multivariate models due to exceeding the tolerance and variance inflation factor (VIF) values specified beforehand, having a close relationship with the variable “Professional training”.

### The relevance domain

The multivariate regression results indicated that EBP training and professional training were significantly associated with the sum score on the *relevance* domain (Table 5). The mean *relevance* score of those who reported having received EBP training was 2.34 higher than those who answered “no” (95% CI = 0.47, 4.21). Regarding professional training, physical therapists had a 5.74 higher mean score (95% CI = 3.28, 8.19) than the assistant nurses (reference). The nurses had a 3.26 higher mean score (95% CI = 1.28, 5.24) than the assistant nurses. The number of years since education was significantly associated with the relevance score in the univariate analysis but did not remain significant in the multivariate model.

**Table 5:** Estimated assosiations between different bakcground variables and mean sum score on the relevance domain (*n* = 291)

Background variables	Unadjusted estimates			Adjusted estimates (*R*^2^ = 0.11)			
	B	95% CI	*p*-value	B	95% CI	*p*-value	β
EBP-training (No [ref] /yes)	3.89	2.30, 5.48	< 0.001	2.34	0.47, 4.21	0.014	0.16
**Level of education** (< bachelor degree [ref] / > bachelor degree)	4.74	2.92, 6.57	< 0.001				
**Years since eduaction** ( 0–5 years [ref] / over 6 years	-2.48	-4.12, -0.84	0.003	-1.41	-3.25, 0.43	0.13	-0.09
**Professional training** (Assistant nurses [ref])							
Physical therapists	7.15	4.95, 9.34	< 0.001	5.74	3.28, 8.19	< 0.001	0.32
Occupational therapists	2.97	0.37, 5.57	0.025	1.81	-1.02, 4.65	0.70	0.08
Nurses	4.08	2.20, 5.95	< 0.001	3.26	1.28, 5.24	0.001	0.22
**Age** (years)	-0.05	-0.13, 0.02	0.15	0.024	-0.05, 0.10	0.55	0.038

B = Regression coefficient

β = Standardsized coefissient Beta

*P* ≤ 0.05 = statistically significant association

Min-max scale values = 14–70

“Professional training” were removed from adjusted model due to multicollinearity

### The terminology domain

The multivariate regression results indicated that EBP training and professional training were significantly associated with the sum score on the *terminology* domain (Table 6). The mean *terminology* score of those who reported having received EBP training was 8.78 higher than those who answered “no” (95% CI = 5.03, 12.52). The only significant association found regarding professional training was that physical therapists had a 12.36 higher mean score (95% CI = 7.42, 17.49) than the assistant nurses (reference). As in the *relevance* domain, the number of years since education was significantly associated with the *terminology* score in the univariate analysis but did not remain significant in the multivariate model.

**Table 6:** Estimated assosiations between different bakcground variables and mean sum score on the terminology domain (*n* = 291)

Background variables	Unadjusted estimates			Adjusted estimates (*R*^2^ = 0.21)			
	B	95% CI	*p*-value	B	95% CI	*p*-value	β
EBP-training (No [ref] /yes)	12.05	8.87,15.24	< 0.001	8.78	5.03, 12.52	< 0.001	0.28
**Level of education** (< bachelor degree [ref] / > bachelor degree	12.42	8.68, 16.16	< 0.001				
**Years since eduaction** ( 0–5 years [ref] / over 6 years	-6.41	-9.83, -2.99	< 0.001	-1.47	-5.17, 2.22	0.43	-0.05
**Professional training** (Assistant nurses [ref])							
Physical therapists	16.97	12.44, 21.5	< 0.001	12.36	7.42, 17.29	< 0.001	0.33
Occupational therapists	5.93	0.56, 11.30	0.03	-0.20	-5.88, 5.49	0.95	-0.004
Nurses	6.02	2.16, 9.89	0.002	3.22	-0.75, 7.19	0.11	0.10
**Age** (years)	-0.24	-0.39, -0.082	0.003	-0.03	-0.02, 0.13	0.70	-0.02

B = Regression coefficient

β = Standardsized coefissient Beta

*P* ≤ 0.05 = statistically significant association

Min-max scale values = 17–85

“Professional training” were removed from adjusted model due to multicollinearity

### The confidence domain

The multivariate regression results indicated that EBP training and the number of years since education were significantly associated with the sum score on the *confidence* domain (Table 7). The mean *confidence* score of those who reported having received EBP training was 4.24 higher than those who answered “no” (95% CI = 1.86, 6.63)). Those who reported that it was more than six years since they finished their education had a 3.27 lower mean score than those who reported “0–5 years since education” (95% CI = -5.66, -0.90)). The professional training variable was significantly associated with the confidence score in the univariate analysis but did not remain significant in the multivariate model.

**Table 7:** Estimated assosiations between different bakcground variables and mean sum score on the confidence domain (*n* = 289)

Background variables	Unadjusted estimates			Adjusted estimates (*R*^2^ = 0.12)			
	B	(95% CI)	*p*-value	B	95% CI	*p*-value	β
EBP-training (No [ref] /yes)	5.80	3.8, 7.8	< 0.001	4.24	1.86, 6.63	< 0.001	0.23
**Level of education** (< bachelor degree [ref] / > bachelor degree	2.47	0.07, 8.87	0.044				
**Years since eduaction** ( 0–5 years [ref] / over 6 years	-5.58	-7.61, -3.56	< 0.001	-3.27	-5.66, -0.90	0.007	-0.17
**Professional training** (Assistant nurses [ref])							
Physical therapists	3.78	0.84, 6.7	0.012	1.01	-2.14, 4.16	0.53	0.04
Occupational therapists	0.33	-3.15, 3.82	0.85	-2.88	-6.51, 0.74	0.19	-0.10
Nurses	2.55	0.04, 5.06	0.046	0.40	-2.14, 2.94	0.76	0.02
**Age** (years)	-0.16	-0.25, -0.06	< 0.001	-0.05	-0.15, 0.05	0.36	-0.06

B = Regression coefficient

β = Standardsized coefissient Beta

*P* ≤ 0.05 = statistically significant association

Min-max scale values = 11–55

“Professional training” were removed from adjusted model due to multicollinearity

### The practice domain

The multivariate regression results indicated that professional training was the only background variable significantly associated with the sum score on the *practice* domain (Table 8). Physical therapists had a 3.94 higher mean score (95% CI = 1.88, 6.00) than the assistant nurses (reference). The nurses had a 1.88 higher mean score (95% CI = 0.22, 3.54) than the assistant nurses. EBP training, number of years since education, and age were not significantly associated with the *practice* score in the univariate analyses or in the multivariate regression model.

**Table 8:** Estimated assosiations between different bakcground variables and mean sum score on the practice domain (*n* = 290)

Background variables	Unadjusted estimates			Adjusted estimates (*R*^2^ = 0.03)			
	B	(95% CI)	*p*-value	B	95% CI	*p*-value	β
EBP-training (No [ref] /yes)	0.80	-0.51, 2.11	0.23	-0.44	-1.99, 1.13	0.58	-0.04
**Level of education** (< bachelor degree [ref] / > bachelor degree	2.15	0.65, 3.65	0.005				
**Years since eduaction** ( 0–5 years [ref] / over 6 years	-0.98	-2.30, 0.34	0.146	-1.13	-2.66, 0.42	0.15	-0.10
**Professional training** (Assistant nurses [ref])							
Physical therapists	3.59	1.78, 5.41	< 0.001	3.94	1.88, 6.00	< 0.001	0.28
Occupational therapists	0.64	-1.5, 2,78	0.56	1.03	-1.35, 3.40	0.40	0.06
Nurses	2.07	0.52, 3.62	0.009	1.88	0.22, 3.54	0.03	0.16
**Age** (years)	-0.02	-0.08, 0.04	0.55	0.02	-0.04, 0.09	0.52	0.04

B = Regression coefficient

β = Standardsized coefissient Beta

*P* ≤ 0.05 = statistically significant association

Min-max scale values = 9–45

“Professional training” were removed from adjusted model due to multicollinearity

### The Sympathy domain

The multivariate regression results indicated that professional training was the only background variable significantly associated with the sum score on the *sympathy* domain (Table 9). The only significant association found regarding professional training was that physical therapists had a 2.48 higher mean score (95% CI = 1.20, 3.75) than the assistant nurses (reference). Similar to the results in the *practice* domain, none of the other background variables were significantly associated with the *sympathy* scores.

**Table 9:** Estimated assosiations between different bakcground variables and mean sum score on the sympathy domain (*n* = 291)

Background variables	Unadjusted estimates			Adjusted estimates (*R*^2^ = 0.04)			
	B	(95% CI)	*p*-value	B	95% CI	*p*-value	β
EBP-training (No [ref] /yes)	0.49	-0.33, 1.31	0.24	0.16	-0.82, 1.13	0.75	0.02
**Level of education** (< bachelor degree [ref] / > bachelor degree	1.47	0.53, 2.40	0.002				
**Years since eduaction** ( 0–5 years [ref] / over 6 years	0.021	-0.81, 0.86	0.96	-0.09	-0.87, 1.05	0.86	0.01
**Professional training** (Assistant nurses [ref])							
Physical therapists	2.24	1.1, 3.38	< 0.001	2.48	1.20, 3.75	< 0.001	0.28
Occupational therapists	0.65	-0.70, 2.0	0.34	0.73	-0.74, 2.20	0.33	0.07
Nurses	0.19	-0.79, 1.16	0.71	0.17	-0.86, 1.20	0.75	0.02
**Age** (years)	0.02	-0.02, 0.06	0.29	0.04	-0.005, 0.08	0.08	0.11

B = Regression coefficient

β = Standardsized coefissient Beta

*P* ≤ 0.05 = statistically significant association

Min-max scale values = 7–35

“Professional training” were removed from adjusted model due to multicollinearity

## Discussion

The aim of this study was twofold. Firstly, we aimed to map EBP knowledge, attitudes, behavior, and self-efficacy among primary healthcare professionals working with older people in the municipal health service in Norway. Secondly, we aimed to examine the associations between the healthcare professionals’ background characteristics and self-reported knowledge, attitudes, self-efficacy, and behavior related to EBP, as measured by the EBP^2^-N. The total sample scored the highest in the *relevance* domain, indicating positive attitudes toward EBP. Relative to the high score on the *relevance* domain, the total sample scored lower on all other domains, indicating a low understanding of research terms, low self-efficacy in performing EBP activities, a lack of perceived compatibility of EBP with professional work, and a low degree of EBP behavior. Further, we found statistically significant differences in mean scores between the professions in all domains except for *confidence*, with the most considerable differences in the *terminology* domain. Across the domains, there was a tendency for physical therapists to score the highest and assistant nurses to score the lowest. Concerning associations, the multivariate regression results indicated that having received EBP training was associated with more positive attitudes, better understanding of common research terms, and higher self-efficacy in performing EBP activities. Additionally, the professional training of the participants was associated with their self-reported EBP attitudes, their understanding of common research terms, their EBP behavior, and their perceived compatibility of EBP with their professional work. Finally, the results suggested that self-efficacy in performing EBP activities declined when more than six years had passed since their education.

### EBP attitudes

All the primary healthcare professionals included in our study generally held positive attitudes toward EBP, as indicated by high scores on the *relevance* domain. Our results were similar to results from previous surveys conducted in Norway, which also found positive attitudes toward EBP [22, 24, 25]. Based on the results of an umbrella review that included systematic reviews from worldwide, positive attitudes toward EBP across professions also seem to be the case internationally [8]. Possible explanations for the positive EBP attitudes in our sample may be related to the Norwegian authorities’ year-long extensive focus on ensuring that Norwegian healthcare is evidence-based [18–21]. In addition, since the participants who had received EBP training scored significantly higher on the *relevance* domain, it seems that being exposed to EBP training is associated with more positive attitudes toward EBP. However, the nature of our cross-sectional design makes it impossible to conclude whether the relationship between EBP training and attitudes is causal and, thus, whether the attitudes are “caught or taught” [2, 43]. Although all professions scored relatively high in the relevance domain, between 55 and 63 on a scale ranging from 14 to 70, the post hoc analyses showed significant differences between professions’ mean scores in this domain. A tendency for physical therapists and nurses to score significantly higher than assistant nurses was also confirmed in the multivariate regression analysis when adjusted for the other background variables and age. While recognizing this tendency of varying attitudes between professions, it is important to emphasize that all professions scored relatively high on the relevance domain, indicating that the attitudes differed only between “positive” and very positive”.

The total sample mean score on the *sympathy* domain was 21.2 on a scale ranging from 7 to 35. This score indicated that the participants perceived EBP as only partly compatible with their professional work. Relative to the results of the *relevance* domain, the lower score on the *sympathy* domain shows that having positive attitudes toward EBP does not necessarily mean that EBP is perceived as compatible with professional work. Our results on the *sympathy* domain were quite similar to those of former studies that used the same instrument [16, 22], showing that our finding is not unique to our sample. In comparison, the mean score of Norwegian bachelor students was 21.8 [22], whereas Australian physical therapists in their second year of working scored 23.0 [16].

### EBP knowledge

The total sample score on the *terminology* domain was low (44.5 on a scale ranging from 17 to 85), indicating that primary healthcare professionals in our study perceived their understanding of common research terms as low. In comparison, bachelor’s students in different professions in the study of Snibsøer et al. (2018) scored 47.0 in the same domain [22]. Our score was also clearly lower when compared to results from a survey among second-year working physical therapists in Australia, who scored 62.2 [16]. A low perceived understanding of research terms is concerning, as knowledge of different terms related to study design, measures of effect, and measures of uncertainty are examples of core competencies required for conducting the steps of EBP [10]. The increasing availability of pre-appraised evidence has made it possible for clinicians to practice EBP without having in-depth knowledge of research terms related to critical appraisal [10]. Still, they have to know how to interpret research results and be able to evaluate the trustworthiness of the evidence they intend to apply [10].

The results of the ANOVA and post hoc tests showed that physical therapists had significantly higher mean scores than all the other professions in the *terminology* domain, and the most considerable difference was found between physical therapists and assistant nurses, with physical therapists scoring 19.50 (95% CI 13.4–25.6) higher. This is not unique to our study. A study by McEvoy et al. (2010) also found that Australian physical therapists scored the highest in this domain compared to participants from other professions such as occupational therapy, medical radiation, and human movement [44]. The difference between physical therapists and assistant nurses remained significant even after adjusting for the other background variables in the multivariate regression. Post hoc tests showed that nurses and occupational therapists also had higher mean scores than assistant nurses, but these differences did not remain significant after adjustment in the multivariate regression. Reasons why physical therapists were the only profession remaining associated with a higher terminology score than assistant nurses in the adjusted estimates and why they scored higher than both nurses and occupational therapists, remain speculations. Potential differences in these groups’ education, curriculum, or professional practice are unknown and warrant further study.

Professional training was not the only background variable associated with the score in the terminology domain. Our multiple regression results also indicated that being exposed to EBP training was associated with a better understanding of common research terms. This result suggests that EBP training may impact perceived EBP knowledge, regardless of a clinician’s professional training.

In a previous study, where we tested the content validity of the EBP^2^-N, several participants expressed concerns about the terminology domain as some of the most specific research terms were perceived as irrelevant [37]. In addition, since this domain includes only items related to research evidence and no other aspects of EBP, such as clinical expertise and patient preferences, it does not capture clinicians’ total understanding of the EBP concept. Consequently, our results related to this domain should be interpreted with caution.

### EBP self-efficacy

The total sample score was also low in the *confidence* domain, indicating that the included primary healthcare professionals in our study have relatively low self-efficacy in performing EBP activities, such as formulating questions, searching, and critical appraisal. Our sample mean score of 31.2 on a scale ranging from 11 to 55 was lower than that of former studies using the same instrument [16, 22]. In comparison, the mean score of Norwegian bachelor students was 34.8 [22], whereas Australian physical therapists in their second year of working scored 42.5 [16]. The low perceived self-efficacy in our sample could be related to the time that had passed since they finished their education. Two-thirds of our sample (61%) graduated more than six years ago, and half (53%) graduated more than eleven years ago. As such, our sample differed from the sample in the two other studies that included undergraduate healthcare students and physical therapists who had worked for only two years [16, 22]. The results of our regression analysis support the assumption that self-efficacy may decrease over time, as those who graduated over six years ago reported a significantly lower confidence score compared to those who graduated 0 to 5 years ago. In a study by McEvoy et al. (2010), the authors discussed that the level of self-efficacy might be masked by “a lack of awareness of limitations in skills in early years of training” and, on the other side, “a lack of acknowledgment of advancement in skills in the later years” [44]. One could only speculate whether the decrease in self-efficacy over time in our sample may be related to a similar phenomenon. At the same time, our regression analysis showed that having received EBP training was significantly associated with a higher score on the confidence domain, showing that the self-efficacy still may be positively affected. Lastly, the *confidence* domain was the only domain without significant differences between professions mean scores. The lack of differences in self-efficacy was somewhat surprising, given that we found substantial differences in perceived knowledge about research terms. However, McEvoy et al. (2010) [44] also reported the same tendency of similar confidence scores between professions, regardless of differences in the perceived knowledge, showing that higher levels of perceived knowledge do not necessarily translate into higher self-efficacy.

### EBP behavior

The total sample mean score in the *practice* domain was 22.2 on a scale ranging from 9 to 45, and the lowest score of all domains. The low score indicated that the included primary healthcare professionals had low levels of EBP behaviors, such as searching for evidence, appraisal, and implementing research into clinical practice in their clinical workday. While a low level of EBP practice is concerning, this is not unique to our sample. Although scoring slightly higher than our sample, the bachelor students in the study by Snibsøer et al. and the second-year working physical therapists in the study by McEvoy et al. scored relatively low with 23.8 and 25.0, respectively [16, 22].

Further, professional training was the only background variable associated with the *practice* score in the regression analysis. As in the *terminology* domain, the regression results showed a tendency of physical therapists and nurses to score significantly higher than assistant nurses. The largest difference in mean scores was found between physical therapists and assistant nurses when adjusted for the other background variables. However, the multivariate regression results showed that physical therapists and nurses scored only 3.94 and 1.88 higher than the assistant nurses. Even the highest score on this domain of 24.1 (physical therapists) could be regarded as a relatively low score on a scale ranging from 9 to 45. Accordingly, the considerable differences found in the *terminology* domain, expressing differences in perceived EBP knowledge, did not seem to translate into a similar difference in EBP behavior.

An interesting finding was that having received EBP training was not associated with any higher score in the *practice* domain. These results align with a recent randomized controlled trial from Koota et al., which evaluated the effectiveness of a multifaceted educational EBP training program in providing basic EBP competencies to hospital nurses [45]. Their results regarding EBP behavior showed that the intervention group improved EBP behavior six months after the program, but after a year, the behavior decreased to the baseline level [45]. However, in our study, it remains unclear whether such a phenomenon, where an initial increase in EBP behavior is followed by a decrease one year after EBP training, may explain the lack of association between EBP training and EBP behavior. This is because of our cross-sectional design, which makes it impossible to draw conclusions about potential changes in EBP behavior over time. Lastly, since we do not know the details of the actual content of the EBP training our participants reported receiving, we do not know whether the lack of association with EBP behavior in any way was related to the training.

### Strength and limitations

Possible limitations that could introduce bias into the results should be acknowledged. Some limitations have already been mentioned in the discussion of the results, such as the risk of measurement bias related to the uncertainties regarding the content validity of the *terminology* subscale.

The sample size of one of our included subgroups included in the one-way ANOVA analysis, the occupational therapists, was lower than the required number to detect differences (Delta 0.3–0.5) in mean score between professions. While we included 38 occupational therapists, the power analysis indicated that at least 64 participants should have been included (sig 5%, power 80%). Therefore, the parts of the one-way ANOVA and regression results related to the occupational therapists should be interpreted with caution.

Our study used a non-random snowball sampling method, as no complete list of all Norwegian primary healthcare professionals exists. While our sampling method may have introduced bias, this approach to recruitment was the most adequate option available. In addition, it was not possible to keep track of the precise number of participants who received the invitation, which impeded the determination of a response rate and hindered us from concluding whether the final sample adequately represented the target population of our study. Further, non-response bias may have occurred, as no information was available for those who received our survey but chose not to respond. For instance, we cannot rule out the possibility that those who agreed to respond to a survey about EBP held more positive attitudes to EBP than the ones who decided not to respond. However, as 59.1% of our sample answered “no” to whether they had received any formal EBP training, it seems reasonable to suggest that our participants at least were not more interested in or well equipped to practice EBP compared to other clinicians in our population. However, this remains a speculation, and our results should be interpreted cautiously regarding generalizability.

Lastly, the instrument used in this study is self-reported, which some considered less favorable than objective measures due to possible limitations with the self-report format [12]. Although self-reported instruments are considered prone to recall and social desirability bias, their use has been encouraged in circumstances where pragmatic options are needed, and objective measurements require more time and human resources [46].

### Implications

Our study contributes to understanding why strategies aimed at changing clinicians’ behavior to practice the steps of EBP more effectively should be evaluated by assessing factors such as EBP knowledge, attitudes toward EBP, self-efficacy, and EBP behavior. Our results are particularly relevant for primary healthcare professionals in municipal healthcare, as the international and national focus is on ensuring healthcare services are evidence-based to provide the highest quality of care to patients and users. The results have several practical implications. While positive attitudes toward EBP were observed in this study, we did not find equally high scores for self-reported knowledge, self-efficacy, or EBP behavior. Thus, our findings support earlier studies that found positive attitudes alone are insufficient to ensure EBP behavior in clinical practice [47]. Furthermore, our results suggest that EBP training should be an integrated part of any strategy designed to promote EBP adoption, as it is associated with improved knowledge about research terminology and higher self-efficacy in performing EBP-related activities. When integrating EBP training as part of a strategy to promote EBP in clinical practice, it is essential to recognize the varying levels of attitudes and knowledge across different professions and adapt the intensity and level of training accordingly. The underlying reasons why different professions seem unequally prepared for learning and implementing EBP should be further studied.

## Conclusion

Our study shows that Norwegian primary healthcare professionals hold positive attitudes toward EBP. However, they also report a low understanding of research terms, low self-efficacy in performing EBP activities, a lack of perceived compatibility of EBP with professional work, and a low frequency of EBP behavior. Further, we found differences between the included professions in four out of five domains, indicating that different professions may be unequally prepared for EBP. Lastly, our results show possible positive consequences of EBP training, as having received EBP training was associated with more positive attitudes, better understanding of common research terms, and higher self-efficacy in performing EBP activities. Having received EBP training was not associated with their self-reported EBP behavior.

## Supplementary Information

Additional file 1. Characteristics of sub-groups.

Additional file 2. Strobe checklist.

Additional file 3. Total sample response frequences per item.

## Data Availability

The datasets used and analyzed during the current study are available from the corresponding author on reasonable request.

## Abbreviations

EBP: Evidence-based practice
EBP^2^: The Evidence-based practice profile questionnaire
EBP^2^-N: The Norwegian version of the Evidence-based practice profile questionnaire
SIKT: The Norwegian Agency for Shared Services in Education and Research
