# Supplementary material for: Knowledge, attitudes, behavior, and self-efficacy related to evidence-based practice among healthcare professionals working in the municipal healthcare service in Norway: a cross-sectional survey
Source: BMC Health Serv Res. 2024 Oct 15;24:1235. doi: 10.1186/s12913-024-11723-4 (PMC11476601; doi:10.1186/s12913-024-11723-4)
Supplement: Supplementary file 1 — Additional file 1. Characteristics of sub-groups. [file 12913_2024_11723_MOESM1_ESM.pdf]

Additional file 1.
Characteristics of sub-groups **(n= 313)**

**Profession (n=313)**	**Occupational therapist (n = 38)**	**Physical therapist (n = 64)**	**Nurses (n = 119)**	**Assistant nurses (n = 74)**	**Medical doctors (n = 3)**	**Other (n = 15)**
**Age (years)** (n=291) Median (min- max)	36 (23 – 66)	37 (23 – 60)	41 (23 – 63)	48.5 (21 – 66)	61 (33 – 72)	43 (31 – 61)
**Level of education n (%)**						
Below upper secondary education	0	0	0	1 (1.4)	0	0
Upper secondary education	0	0	0	31 (41.9)	0	2 (13.3)
Tertiary vocational education	0	0	1 (0.8)	40 (54.1)	0	0
Bachelor's Degree	35 (92.1)	51 (79.7)	100 (84)	2 (2.7)	0	12 (80)
Master's Degree	3 (7.9)	12 (18.8)	18 (15.1)	0	3 (100)	1 (6.7)
PhD	0	1 (1.6)	0	0	0	0
**Years since education n (%)**						
0-5 years	18 (47.4)	27 (42.2)	48 (40.3)	24 (32.4)	0	4 (26.7)
6- 10 years	6 (15.8)	15 (23.4)	15 (12.6)	15 (20.3)	2 (66.7)	4 (26.7)
11-15 years	7 (18.4)	8 (12.5)	20 (16.8)	11 (14.9)	0	4 (26.7)
16-20 years	3 (7.9)	5 (7.8)	11 (9.2)	5 (6.8)	0	0
21-25 years	1 (2.6)	6 (9.4)	13 (10.9)	8 (10.8)	0	2 (13.3)
26-30 years	1 (2.6)	3 (4.7)	7 (5.9)	5 (6.8)	0	1 (6.7)
Over 30 years	2 (5.3)	0	5 (4.2)	6 (8.1)	1 (33.3)	0
**Clinical work experience (n = 313)**						
0-5 years	15 (39.5)	16 (25.0)	28 (23.5)	7 (9.5)	1 (33.3)	1 (6.7)
6- 10 years	6 (15.8)	21 (32.8)	26 (21.8)	17 (23.0)	0	3 (20.0)
11-15 years	7 (18.4)	7 (10.9)	20 (16.8)	7 (9.5)	0	5 (33.3)
16-20 years	2 (5.3)	10 (15.6)	13 (10.9)	13 (17.6)	0	0
21-25 years	3 (7.9)	6 (9.4)	13 (10.9)	9 (12.2)	0	3 (20.0)
26-30 years	2 (5.3)	3 (4.7)	6 (5.0)	6 (8.1)	0	2 (13.3)
Over 30 years	3 (7.9)	1 (1.6)	13 (10.9)	15 (20.3)	2 (66.7)	1 (6.7)
**EBP experience/ training (no)**	11 (28.9)	24 (37.5)	73 (61.3)	64 (86.5)	2 (66.7)	11 (73.3)
**Yes**	27 (71.1)	40 (62.5)	46 (38.7)	10 (13.5)	1 (33.3)	4 (26.7)
1-3 hours	3 (11.5)	7 (17.9)	4 (8.9)	0	0	0
3-10 hours	9 (34.6)	13 (33.3)	19 (42.2)	2 (22.2)	0	2 (50.0)
10-20 hours	6 (23.1)	8 (20.5)	8 (17.8)	4 (44.4)	0	0
Over 20 hours	8 (30.8)	11 (28.2)	14 (31.1)	3 (33.3)	1 (100)	2 (50.0)

Other= social educators, assistants, leaders
* =
