# Supplementary material for: Knowledge, attitudes, behavior, and self-efficacy related to evidence-based practice among healthcare professionals working in the municipal healthcare service in Norway: a cross-sectional survey
Source: BMC Health Serv Res. 2024 Oct 15;24:1235. doi: 10.1186/s12913-024-11723-4 (PMC11476601; doi:10.1186/s12913-024-11723-4)
Supplement: Supplementary file 3 — Additional file 3. Total sample response frequences per item. [file 12913_2024_11723_MOESM3_ESM.pdf]

Additional file 2

Total sample response frequences per item in the EBP^{2}-N

Domain/ items	Response options				
**Relevance**	Not true at all	Not really true	Possibly true	Quite likely true	Very true
Item 1 (n = 313)	0.3 %	1.6 %	1.6 %	32.3 %	54 %
Item 2 (n = 313)	1.3 %	3.8 %	15 %	32.6 %	47.3 %
Item 3 (n = 313)	0.6 %	4.2 %	24.6 %	40.6 %	30 %
Item 4 (n = 313)	3.2 %	15.7 %	34.2 %	32.9 %	14.1 %
	No intention at all	Unlikely to consider doing it	Could consider doing it	Highly likely to consider doing it	Absolutely intend to do it/ jeep doing it
Item 5 (n = 313)	0.6 %	0.6 %	17.3 %	35.1 %	46.3 %
Item 6 (n = 313)	0.3 %	2.2 %	15.7 %	33.9 %	47.9 %
Item 7 (n = 313)	0	1.6 %	12.8 %	28.1 %	57.5 %
Item 8 (n = 313)	0.3 %	1.3 %	5.8 %	29.1 %	63.6 %
	Strongly Disagree	Disagree	Neutral	Agree	Strongly agree
Item 9 (n = 313)	0	0	7.7 %	43.5 %	48.9 %
Item 10 (n = 313)	0.6 %	1.9 %	12.1 %	52.7 %	32.6 %
Item 11 (n = 313)	0.6 %	1.0 %	13.4 %	55.3 %	29.7 %
Item 12 (n = 313)	0.6 %	0.3 %	7.0 %	48.2 %	43.8 %
Item 13 (n = 313)	0	0	13.4 %	48.6 %	38.0 %
Item 14 (n = 313)	0.3 %	0.6 %	12.5 %	47.9 %	38.7 %
**Sympathy** (reversed scored)	Strongly Disagree	Disagree	Neutral	Agree	Strongly agree
Item 15 (n = 313)	13.7 %	33.5 %	44.7 %	7.3 %	0.6 %
Item 16 (n = 313)	0.3 %	2.6 %	24.6 %	49.8 %	22.7 %
Item 17 (n = 313)	1.6 %	6.4 %	33.2 %	47.3 %	11.5 %
Item 18 (n = 313)	5.1 %	34.5 %	41.5 %	17.3 %	1.6 %
Item 19 (n = 313)	5.4 %	28.8 %	47.0 %	18.2 %	2.6 %
Item 20 (n = 313)	6.4 %	20.4 %	30.4 %	37.7 %	5.1 %
Item 21 (n = 313)	14.7 %	45.0 %	24.3 %	14.4 %	1.6 %
**Terminology**	Newer heard the term	Have heard it but don't understand	Have some understanding	Understand quit well	Understand and could explain to others
Item 22 (n = 313)	14.1 %	8.3 %	39.3 %	31.6 %	6.7 %
Item 23 (n = 313)	14.7 %	8.9 %	36.1 %	30.0 %	10.2 %
Item 24 (n = 313)	12.1 %	7.3 %	24.9 %	30.7 %	24.9 %
Item 25 (n = 313)	37.1 %	19.5 %	28.8 %	10.9 %	3.8 %
Item 26 (n = 313)	13.7 %	22.4 %	29.4 %	22.4 %	12.1 %
Item 27 (n = 313)	40.3 %	12.1 %	25.6 %	16.6 %	5.4 %
Item 28 (n = 313)	38.7 %	17.9 %	23.0 %	16.6 %	3.8 %
Item 29 (n = 313)	33.5 %	15.7 %	26.2 %	16.6 %	8.0 %
Item 30 (n = 313)	68.7 %	14.1 %	12.1 %	3.5 %	1.6 %
Item 31 (n = 313)	41.2 %	13.4 %	29.7 %	13.4 %	2.2 %
Item 32 (n = 313)	20.8 %	11.2 %	27.8 %	25.6 %	14.7 %
Item 33 (n = 313)	24.6 %	15.3 %	30.4 %	20.8 %	8.9 %
Item 34 (n = 313)	12.8 %	9.3 %	30.7 %	31.9 %	15.3 %

Item 35 (n = 313)	20.4 %	7.0 %	23.6 %	22.0 %	26.8 %
Item 36 (n = 313)	62.6 %	13.7 %	15.3 %	5.4 %	2.9 %
Item 37 (n = 313)	37.4 %	20.8 %	26.8 %	11.8 %	3.2 %
Item 38 (n = 313)	34.2 %	14.1 %	30.7 %	16.6 %	4.5 %
**Practice**	Never	Monthly or less	Fortnightly	Weekly	Daily
Item 39 (n = 312)	23.1 %	47.1 %	8.7 %	16.7 %	4.5 %
Item 40 (n = 312)	27.2 %	59.3 %	7.1 %	4.8 %	1.6 %
Item 41 (n = 312)	31.4 %	51.0 %	8.3 %	8.3 %	1.0 %
Item 42 (n = 312)	30.4 %	51.6 %	7.4 %	7.7 %	2.9 %
Item 43 (n = 312)	12.5 %	44.6 %	10.9 %	20.5 %	11.5 %
Item 44 (n = 312)	3.2 %	8.0 %	5.8 %	33.7 %	49.4 %
Item 45 (n = 312)	5.4 %	58.3 %	21.5 %	13.5 %	1.3 %
Item 46 (n = 312)	12.2 %	55.8 %	12.5 %	14.7 %	3.8 %
Item 47 (n = 312)	26.0 %	52.9 %	8.3 %	10.6 %	2.2 %
**Confidence**	Not at all confident	A little confident	Reasonably confident	Quite confident	Very confident
Item 48 (n = 311)	46.9 %	26.0 %	16.1 %	8.0 %	2.9 %
Item 49 (n = 311)	0.3 %	10.0 %	26.4 %	31.5 %	31.8 %
Item 50 (n = 311)	1.3 %	10.3 %	34.4 %	38.3 %	15.8 %
Item 51 (n = 311)	17.0 %	36.7 %	25.4 %	15.8 %	5.1 %
Item 52 (n = 311)	11.6 %	27.0 %	28.6 %	25.1 %	7.7 %
Item 53 (n = 311)	10.0 %	24.4 %	28.0 %	27.3 %	10.3 %
Item 54 (n = 311)	18.0 %	30.9 %	22.8 %	21.5 %	6.8 %
Item 55 (n = 311)	30.2 %	27.7 %	23.5 %	12.5 %	6.1 %
Item 56 (n = 311)	13.2 %	35.4 %	26.4 %	19.0 %	6.1 %
Item 57 (n = 311)	17.7 %	33.1 %	25.4 %	19.0 %	4.8 %
Item 58 (n = 311)	6.8 %	28.0 %	35.0 %	22.5 %	7.7 %
**Non domain items**	Strongly Disagree	Disagree	Neutral	Agree	Strongly agree
Item 59 (n = 311)	10.6 %	26.7 %	32.2 %	25.7 %	4.8 %
Item 60 (n = 312)	15.1 %	24.0 %	20.5 %	27.9 %	11.9 %
Item 61 (n = 311)	15.4 %	24.1 %	22.5 %	26.0 %	11.9 %
Item 62 (n = 313)	2.2 %	10.2 %	22.0 %	39.3 %	26.2 %
Item 63 (n = 312)	27.6 %	33.3 %	23.4 %	11.9 %	3.8 %
Item 64 (n = 313)	2.9 %	12.5 %	35.1 %	39.9 %	9.6 %
Item 65 (n = 313)	4.8 %	9.6 %	25.2 %	44.4 %	16.0 %
Item 66 (n = 313)	4.8 %	8.0 %	34.5 %	39.0 %	13.7 %
